# Women's needs and expectations in midwifery care – Results from the qualitative MiCa (midwifery care) study. Part 1: Preconception and pregnancy

**DOI:** 10.1016/j.heliyon.2024.e25862

**Published:** 2024-02-07

**Authors:** Toni Maria Janke, Nataliya Makarova, Janne Schmittinger, Caroline Johanna Agricola, Merle Ebinghaus, Christine Blome, Birgit-Christiane Zyriax

**Affiliations:** a Competence Center for Health Services Research in Dermatology (CVderm), Institute for Health Services Research in Dermatology and Nursing (IVDP), University Medical Center Hamburg-Eppendorf, Martinistr. 52, 20246 Hamburg, Germany; b Midwifery Science—Health Care Research and Prevention, Institute for Health Service Research in Dermatology and Nursing (IVDP), University Medical Center Hamburg-Eppendorf, Martinistr. 52, 20246 Hamburg, Germany

**Keywords:** Continuity of midwifery care, Preconception, Pregnancy, Preferences, Qualitative research

## Abstract

Midwifery services play an important role in healthcare provision, birth preparation and prevention.

Knowledge on women's expectations, preferences and needs regarding midwifery care is crucial not only for clinical care during preconception and pregnancy and research, but also for educational purposes. This descriptive qualitative study aimed to investigate the expectations, preferences and the needs of women concerning midwifery care in Germany.

Experienced researcher team conducted interviews with women who have the desire to get pregnant and online focus groups with women in early and late pregnancy. A purposeful recruitment strategy with maximum variation sampling was applied to reach diversity in the sample regarding age, previous children and socioeconomic background.

A total of 26 women participated. In the qualitative content analysis according to Mayring, seven main categories were developed for both preconceptional phase and pregnancy: (a) care by midwife, (b) care by obstetrician, (c) involvement of family, (d) need for information, (e) physical aspects, (f) psychological aspects and (g) orientation in healthcare system. One additional category referenced (h) organisation and bureaucracy in pregnant women. Women appreciated the personalised experience provided by midwives leading to trust and empowerment. Women's experiences with midwifery care varied. They reported contradictory information they received about services and care options. They valued interprofessional cooperation, continuity of care, structured information and personalised counselling.

Midwives play an important role in healthcare provision, birth preparation and prevention.

In order to depict the care situation quantitatively, to personalise care and to optimise healthcare models, a tool to assess the quality of healthcare and to evaluate women's needs and benefits of midwifery care will be developed based on the findings of this study. From the public health perspective, deficits in the German healthcare system concerning insufficient intra-sectoral communication, time pressure and low remuneration should be resolved in further research steps and policy action.

## Highlights

• From the user's perspective, midwifery care is crucial for the preconceptional and pregnancy phases.
• Women appreciated the personalised experience provided by midwives leading to trust and empowerment, reported inequalities in the information they received about services and care options.
• Study participants valued interprofessional cooperation, continuity of care, more structured information and personalised counselling.
• Study findings have been categorised in a more holistic approach combining interprofessional care and involving family expertise, including physical and psychological aspects and highlighting the role of the midwife as a key contact person within the continuity of care.

## 1 Introduction

Midwives play an important role in healthcare provision, birth preparation and prevention. They are important providers in healthcare, supporting women and families along the entire continuity of care [1]. Continuity of care is a concept rooted in the primary care of an individual by the same provider over time [2,3]. Midwifery continuity of care includes the provision of care starting with the desire to have children, followed by pregnancy, birth, puerperium and ending with breastfeeding, whereby care is provided by the same midwife or a small team of midwives [4]. In Germany, midwifery care implements a preventive rather than a curative strategy during family planning. Furthermore, the implementation of midwifery continuity of care has been shown to increase satisfaction in pregnant women [5,6]. Women appreciate the personalised experience provided by midwives, leading to trust and empowerment of women [7].

Family planning, the desire to have children and pregnancy are formative periods for women with multi-layered processes of physical and mental change. During this important time, medical and psychosocial care can be provided by midwives and obstetricians. Prenatal care in healthy women with predictable physiological birth can be carried out alternately by midwives and obstetricians or solely by one of these two professional groups. In Germany, prenatal care approaches are based on the guidelines for medical care during pregnancy and after childbirth, developed by the Joint National Committee, an institution of the German Ministry of Health. These guidelines provide the basis for examinations during pregnancy and after childbirth, in particular the scope and timing of the services, and they provide a document to be used for examination results [8]. The implementation of midwifery continuity of care is regulated in the Social Security Statute Book V [8]. Additionally, major parts of healthcare during pregnancy are regulated by obstetricians [8].

Midwifery care for women with the desire to have children can include the discussion of aspects such as physical activity, nutrition, thyroid values, oral health and mental stress. However, such midwifery care and counselling throughout the preconceptional phase is currently rarely provided and the respective competencies of midwives are rarely available or communicated [9].

Midwifery healthcare service faces multiple challenges. On the one hand, a higher age of first-time mothers, an increased prevalence of chronic diseases and higher rates of multimorbidity pose increasing complexity in provision of care during pregnancy, birth and puerperium in Germany [10]. On the other hand, there has been a lack of midwives for more than a decade in Germany [10]. Moreover, midwifery healthcare provision as well as education in Germany is lacking evidence-based approaches [10]. Therefore, midwifery training was reformed in the European Union (EU) education standard in 2020 [11]. In Germany, university study courses were implemented recently with the aim to educate experts providing evidence-based care throughout all phases of midwifery care in inpatient and outpatient settings [10]. Transferring knowledge and experiences from healthcare practice into the curriculum and back to practice plays a decisive role in developing study contents. Thus, the knowledge based on women's expectations, preferences and needs in regard to midwifery care is crucial not only during preconception and pregnancy and research [12], but also for educational purposes.

In midwifery, woman-centred care as a philosophical and a pragmatic concept has become well known in the last decades [13]. This concept prioritises the woman's individual unique needs (organisational, healthcare-related and pregnancy-specific), as defined by the woman herself, assigns the woman choice, control and continuity of care, emphasises the woman-midwife relationship and thus requires dialogue in line with evidence from qualitative research [13]. With this study, we aimed to investigate expectations as well as preferences and to identify needs of women in regard to midwifery care.

Since midwives' healthcare services, fields of work, legal framework and billing procedures between health insurance companies and midwives throughout preconception and pregnancy differ from country to country within the EU, comparative analyses would not lead to evident results. This makes country-specific research necessary. Therefore, we focused on Germany specific research within midwifery care. The necessity of conducting this study becomes evident in light of the discontinuity in midwifery care and the tailored midwives’ healthcare services within the German healthcare system concerning this vulnerable phase of life, which has impacts and consequences for overall and long-term family health.

In Germany, there is a lack of qualitative studies investigating the experiences and wishes of women regarding midwifery care. The only qualitative study we found in Germany focused on pregnant women and mothers concerning systemic aspects of midwifery [12]. Internationally, we found evidence regarding the quality of maternity care services as experienced by women [14], women's perception of continuity of team midwifery care [15], midwifery-led continuity of care in the birth path [16] and satisfaction with comprehensive women-centred maternity support [17]. In a scoping review Vogels-Broecke [18] and colleagues identified seven dimensions describing women's experiences, but only of the perinatal period [18]. Considering the limited amount of evidence, investigating expectations, preferences and needs of pregnant women in regard to midwifery care in the respective healthcare system using qualitative research methods is essential. We based our overall research strategy on the sequential exploratory design described by Ref. [19], beginning with a qualitative study, using focus groups and interviews for an in-depth understanding of expectations, preferences and needs of women in midwifery care, and analysing data inductively. Based on results of the present study, we plan to quantitatively evaluate the quality of care by developing an instrument with which we intend to facilitate the adequate depiction of the care situation of women and to personalise care. The development of a tool to assess the quality of healthcare and to evaluate women's needs and benefits of midwifery care may be advantageous for healthcare during the whole midwifery continuity of care, including family planning, pregnancy, birth, puerperium, and breastfeeding [3,20].

In our study, qualitative data were collected along the whole midwifery continuity of care. In light of the large amount of study results, we decided to split our study in two parts: (1) preconception and pregnancy (this paper) and (2) childbirth and early parenthood (the companion paper). In the first part of this qualitative study, we aimed to investigate expectations, preferences and needs of women in midwifery care with the desire to have children and during pregnancy.

## 2 Methods

We studied the phases of preconception, pregnancy, birth, puerperium and early parenthood. In the present research, we focus on preconception and pregnancy. Results on childbirth and early parenthood are presented in a contiguous publication.

### 2.1 Ethical aspects and data protection

The study complies with all relevant national regulations, institutional policies and is in accordance with the tenets of the Helsinki Declaration. This study was approved by the Local Psychological Ethics Committee of the University Medical Center Hamburg-Eppendorf (LEPK-0427). All offices were secured with a manual locking system. Only members of the study team had access to the data. The computers for storing, processing and analysing data were cryptographically encrypted and password-protected. The data is stored on the respective protected project drives or folders, to which only authorised study staff have access for ten years. Software and hardware firewalls protect against unauthorised access from outside. Every participant has read and signed informed consent, covering the study participation, processing the data, data protection aspects and publishing the results.

### 2.2 Participants

We included women with the desire to have children (preconception), women in early pregnancy (up to the 24th week of pregnancy) and women in late pregnancy (beyond the 24th week of pregnancy). Participants were required to speak and understand German and to be able to give informed consent. No further inclusion or exclusion criteria were defined.

A purposeful strategy according to maximum variation sampling was applied to achieve diversity in the sample regarding age, previous children and socioeconomic background. Participants were recruited via midwives, social media, personal contacts and a newsletter at the study center, UKE, Hamburg, Germany. The study information, conditions of participation (in this case, desire to have a child, early pregnancy (up to the 24th week of pregnancy) and late pregnancy (beyond the 24th week of pregnancy)), contact address and telephone number of the person responsible for the study (TMJ) were distributed via the channels mentioned above. Information about the financial incentive (€ 20, provided to increase motivation for participation and to appreciate the effort participants made) was also published in advance. During the following month (after the advertisement was distributed), the women interested in participation contacted us via e-mail and telephone. We assembled the groups for interviews and focus groups. The person responsible for the study (TMJ) did not know study participants beforehand. Planning the study, we decided to follow the approach of recruitment until data saturation and estimated that we could reach this with about five interviews in the preconception phase and four focus groups (two with women in early pregnancy and two with women in late pregnancy) with five study participants in every focus group. While the data collection approach left the opportunity open to recruit more participants than our initially estimated number until data saturation, the planned composition of five women per focus group was not attained. This was due to short-notice cancellations by some pregnant women because of illness or unexpected delivery in late pregnancy. Ultimately, we carried out seven focus groups with two to five (3, 2, 3, 4, 3, 4 and 2) participants each in both pregnancy groups. We did not challenge with dropouts within this study. All participants who signed informed consent participated in both the interviews and focus groups. Short-notice cancellations due to illness or unexpected delivery during late pregnancy took place prior to obtaining informed consent signatures.

### 2.3 Study conduct

Two different methods of data collection were used in this study. Pregnant women participated in focus groups [21] as this method allows for fruitful discussions between multiple individuals.

Two researchers (TMJ, NM or ME) were involved in conducting each focus group: one researcher guided the interview (either TMJ or NM), a second researcher was present to document the course of conversation (TMJ, NM or ME). The involvement of two researchers in the data collection was essential in this qualitative research approach to ensure data quality. Women in the preconceptional phase participated in problem-centred one-to-one interviews [22] as this phase can be highly sensitive, especially in women with a long-lasting unsuccessful wish to have children. Interviews were conducted by a scientifically working midwife from our research group, who is active in the counselling of women with the desire to have children, who was aware of the setting, the ethical aspects of the target group and best suited as an interviewer.

The selection of a single researcher for the interviews in our study was based on the mental and psycho-social aspects of this vulnerable group, as well as ethical issues. The involvement of multiple interviewers in the preconception group was not possible. We considered it essential that a midwife who is experienced and currently active in the counselling interviewed this particularly vulnerable group. Only one researcher (JS) in our team met these criteria. She was trained in conducting qualitative interviews and took notes during the interviews to enhance transparency.

**Fig. 1:**
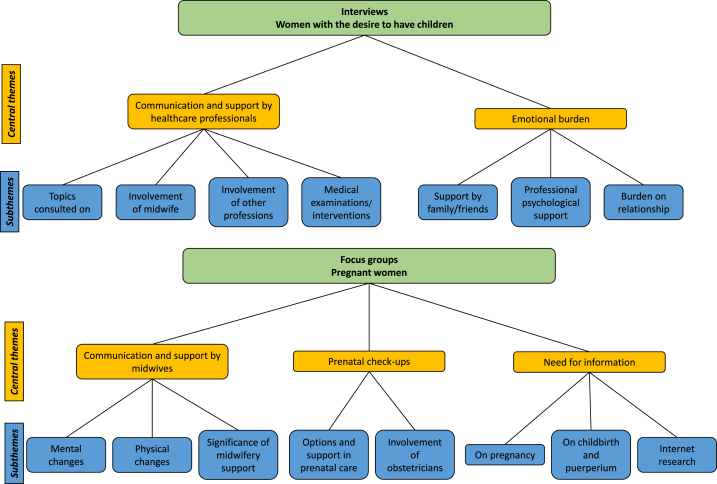
Central themes and subthemes based on interview and focus group guides.

Researchers experienced in qualitative studies (TMJ and NM) prepared and pilot-tested semi-structured interview and focus group guides. These semi-structured guidelines ensured that the most important topics were covered, while at the same time giving the researchers the freedom to dive deeper into new topics raised by participants. Data were assessed between November 2021 and April 2022. As a result of the Covid-19 pandemic, focus groups were conducted at the study center, UKE online via zoom-software and interviews partly face-to-face and partly via phone or zoom. The interviews and focus groups lasted between 1 h to one and half hour, and began with a round of introduction by researchers, their area of research, their role in the research group, and their experience in qualitative research. Fig. 1 summarises central themes and subthemes that were discussed in interviews with women who have the desire to have children and in focus groups with pregnant women.

### 2.4 Data management and analysis

Interviews and focus groups were audio recorded and transcribed verbatim. Two student assistants transcribed the recordings. Two researchers (TMJ, NM) were involved in data analysis. The audio recordings were randomly checked to match the respective transcripts to ensure data quality. Data was analysed using qualitative content analysis according to Mayring [23] with the qualitative data analysis software MAXQDA 2022 (VERBI software, 2021, Berlin, Germany). After familiarisation with the data, content-bearing text passages were paraphrased. Text passages with the same content were subsumed into the same paraphrase. From this, paraphrases were clustered into categories. In an iterative process, categories were then clustered into higher-level categories. Two researchers (TMJ, NM) coded and categorised the data independently to ensure reliability of results. After the independent categorisation process, results were merged and discrepancies discussed. We aimed to reach no more than three to four hierarchical levels of categories to keep the category system clear and manageable. Supplementary Box S1 exemplifies this process, using MAXQDA, as well as the subsequent translation of categories from German to English. New participants were recruited until the category system reached saturation, meaning that no new themes emerged from additional participants.

The whole category system was clustered into the phases of midwifery care. In this process, it was decided to combine the data from the groups in early and late pregnancy. As it was possible that phase-specific groups touched upon topics of other phases, we decided to categorise the data according to the phase that was discussed, not according to the predefined recruited groups.

We adhered to the Consolidated Criteria for Reporting Qualitative Research (COREQ) statement [24]. The completed checklist is provided in Supplementary Table S1.

## 3 Results

Our sample consisted of five women in the preconceptional phase and 21 pregnant women (Table 1). The participants’ mean age was 32.8 years (range: 24–42) and they had between zero and three previous children prior to their current pregnancy. All participants were in a relationship and the mean age of their partners was 34.7 years (range: 27–52). More baseline characteristics of study participants are summarised in Table 1.

**Table 1:** Baseline characteristics of participants.

Variable	M (SD)	Range
Age of woman (years)	32.8 (4.2)	24–42
Age of partner (years)	34.7 (6.1)	27–52
Number of prior children	0.5 (0.7)	0–3
	**n**	**%**
Phase		
Preconception	5	19.2
Early pregnancy (≤24th week)	9	34.6
Late pregnancy (>24th week)	12	46.2
Place of residence (federal state of Germany)		
Baden-Wuerttemberg	2	7.7
Hamburg	14	53.8
Lower Saxony	5	19.2
Schleswig-Holstein	4	15.4
Thuringia	1	3.8

The category system was divided into two clusters, preconception and pregnancy. Despite using two different methods, interviews and focus groups, we aimed to align categories of both clusters, preconception and pregnancy, as much as possible to enable comparability. The final category system with main and subcategories as well as a description for each subcategory are shown in Table 2.

**Table 2:** Category system including the clusters preconception and pregnancy (dark grey) with main categories (light grey) and subcategories (white), as well as a description for each subcategory.

Preconception	Pregnancy	Description
Potential care by midwife	Care by midwife	
	Care at home/in undisturbed environment	Describes the midwife's visits at home during pregnancy
	Availability of the midwife	Being able to reach the midwife for questions/problems
	Midwife important for birth and postpartum	The midwife's role during child birth and postpartum
Competencies of the midwife	Competencies of the midwife	Skills/knowledge that a midwife can offer and her responsibilities as compared to obstetricians
	Continuity in midwifery care	Care provision by the same midwife throughout and beyond pregnancy
	Responsibility and control	Being able to either to maintain the control or to hand over responsibility
Relationship with midwife	Relationship with midwife	The nature and importance of the relationship between woman and midwife
**Care by obstetrician**	**Care by obstetrician**	
	Accessibility of obstetrician	Good or limited accessibility of obstetrician, e.g. if there are health problems during the pregnancy and the pregnant woman should be referred to hospital
Relationship with obstetrician	Relationship with obstetrician	The nature and importance of the relationship between woman and obstetrician
Alternative treatments		Offers being presented beyond conventional medical options
Desire for knowledge transfer by obstetrician		The extent of information provided by the obstetrician
**Involvement of family**	**Involvement of family**	
	Sibling (further children)	Support in preparing previous children to become siblings
Family and friends	Family and friends	Involving family and friends in the processes, information exchange, support by family members and friends
Partner	Partner	Involvement of partner in provided information and care by midwife and obstetrician
**Need for information**	**Need for information**	
	Source of information	Describes the (professional) sources that women get and wish to get information from
Topics and questions	Topics and questions	The issues that arise throughout preconception and pregnancy women wish to talk about
Availability of information	Availability of information	The different ways to receive reliable information and offers by healthcare professionals
	Time of information	The timing of when information is most helpful and best received
**Physical aspects**	**Physical aspects**	
	Improvement of physical complaints	Support in coping with and relieving physical issues
	Need to talk about physical complaints	Being able to address issues with a healthcare professional
	Health problems during the pregnancy	Presence of discomforts and issues going along with pregnancy
Physical changes		e.g. hormonal changes, weight gain etc.
Search for causes		Trying to determine the reasons that cause problems to get pregnant
Side effects		Adverse reactions to fertility treatment
Endometriosis		The role of endometriosis in preconception
**Psychological aspects**	**Psychological aspects**	
	“It'll be alright”	Trusting the process of pregnancy and the body's capabilities
Acknowledging individuality	Acknowledging individuality	Knowing that every person and life processes are different, appreciative intimate communication
Psychological burden	Psychological burden	Emotional strains and worries and professional support in coping with them
	Respect for life with (another) child	Worrying about feasibility and what life will be like with (another) child
	Worries about the child	Concerns regarding the health of the unborn child
	Preparation for birth	Mentally preparing for realities of and wishes for childbirth
	Previous experiences	Role of previous pregnancy experiences in feeling confident in current pregnancy
	Looking forward to child	Anticipation and excitement for life with the child
Exchange with others		Role of support by and exchanging experiences with other affected people
Fluctuation in pursuing wish for child		Changes in mental presence of and focusing on trying to get pregnant
Desire for psychological support		Professional support for mental strains going along with issues with getting pregnant
Unfulfilled desire for child as taboo subject		Negative emotional impact of tabooing and benefits of normalising the subject
**Orientation in healthcare system**	**Orientation in healthcare system**	
Utilisation of examinations and treatments	Utilisation of services and courses	Describes different offers that women had accepted
	Communication within the system	Interprofessional information transfer between care providers attending the same woman
Information about health care providers	Understanding processes	Being aware about the timing of undertaking steps
Referral to fertility center		Timing and organisation of referrals
Private-payer services		Expenses and financial burden going along with fertility treatments
	**Organisation and bureaucracy**	
	Search for midwife	Difficulties in finding an available midwife
	Organisational preparation for birth	Practical arrangements necessary to make for the time of childbirth
	Consideration and registration of birthplace	Evaluating preferences, choosing and registering at preferred place for birth
	Organisation for puerperium	Practical arrangements necessary to make for the puerperium
	Pregnancy and other areas of life	Considering practical impacts pregnancy has on different aspects such as career and travel

### 3.1 Preconception

#### 3.1.1 Potential care by midwife

In the preconceptional phase, women did not know that midwives could serve as care providers or that they could discuss this topic with midwives, except for participants who had midwives as family members or friends. Nevertheless, women asked for an adviser both in an emotional and a professional way as well as someone with a holistic approach. Women wished for a person who offers the time to talk and a trusting and safe environment. At the same time, they recognised that the support of women in the preconceptional phase requires additional training for midwives and that boundaries of competencies need to be acknowledged.

“The doctors are great, they explain, they do a lot, you can ask 17,000 times, but you also have to know which questions to ask. And I miss someone who somehow / in between, like someone where you can come back again and again to sum up, to plan steps, or just to discuss what happened, what makes sense. What else I can do for myself.” (30 years, no previous children)

“I think that if you have a point of contact, a person who knows the subject well and perhaps also has the time that the doctors don't have, that can give you a lot of safety and reduce stress.” (24 years, no previous children)

#### 3.1.2 Care by obstetrician

In gynaecological treatment, women found empathy from obstetricians to be lacking and missed in-depth knowledge transfer. One woman reported that her obstetrician was open for alternative treatment options and provided homoeopathic remedies, which she appreciated.

“Well, I really have to say that with doctors I have, I'm not that satisfied because during all this time I've had a lot of diagnoses just thrown at me or not explained to me at all” (24 years, no previous children)

“For me, it already starts with the ambulatory obstetricians. You already notice massive deficits there. In principle, they can't really help you. They do some kind of cycle monitoring, which they also did for me, where I think to myself ‘unfortunately I have a clue and somehow you don't do it properly’.” (30 years, no previous children)

#### 3.1.3 Involvement of family

Women reported that they discuss their desire to have a child with close family members and friends. They frequently discussed the topic with their partner, but at the same time, this topic could put a strain on the relationship. One woman outlined the importance of having consultation appointments together with their partner, in order for potential examinations and tests to be taken into consideration for both partners from the beginning.

“We as a couple are open about it with a few friends. And with my family but not with my husband's family.” (30 years, no previous children)

“I also notice that there are big differences between male perception and female perception, and my husband says so himself.” (37 years, 2 children)

#### 3.1.4 Need for information

Women emphasised that it is important to receive trustworthy information. Information from different providers might, on the one hand, be more comprehensive, but on the other hand possibly contradictory. The most important information women asked for were a more in-depth explanation of examinations and treatments, which treatment options are available, and to debrief any results. Women would like to be able to clarify questions at the beginning of as well as throughout the preconceptional phase.

“I don't know much about it. I know quite a few women who are in fertility clinics, who also deal with the subject and I don't know any who are satisfied with their treatment. So, with this interpersonal thing, with taking the time, with explaining what is going on and so on. And I think it would be really good to not only have a consultation with the doctors beforehand, but also to have the midwives outside the clinic.” (24 years, no previous children)

“Then, they tell you the result, but the interpretation is missing there, too.” (30 years, no children)

#### 3.1.5 Physical aspects

Endometriosis was the most frequently mentioned condition in the preconceptional phase, meaning that women were either screened for or diagnosed with endometriosis. For many women, this diagnosis led to an increased engagement with the topic of preconception. At the same time, women asked for information about treatment options and the consequences of treatment for their desire to have a child. Another woman reported that her search for issues causing her fertility struggles was dismissed due to past bulimia. Also, side effects from fertility treatments were reported, even affecting women's job performance.

“Unfortunately, the doctors position themselves quite clearly, so when you say you don't have a menstrual cycle and the question comes up, ‘Did you have eating disorders?’ and then you say ‘Yes’ and then ‘Ah. All done.’ So they don't look for anything any further.” (24 years, no previous children)

“I had a laparoscopy almost exactly a year ago and endometriosis was diagnosed. And for that reason alone, I have been more concerned with the whole topic of wanting to have children, because it is simply a disease where it has not yet been scientifically proven, why exactly it leads to infertility. That's why I've looked into it a bit more.” (30 years, no previous children)

#### 3.1.6 Psychological aspects

Having an unfulfilled wish to have children presented a psychological burden to women and many expressed the need to receive psychological support. Women appreciated the opportunity to talk about the topic with other people who had similar experiences. At the same time, they wished the topic to be de-tabooed and normalised. Women also reported that the topic was sometimes more present in their lives than other times.

“Then, you always have a bit of a false expectation of getting pregnant, I think that's always the first problem, that you think you're no longer using contraception and bang you're pregnant (laughs). And for me at least I can say that the first few months were extremely hard, because you always / well exactly, because your brain, I think, just didn't think it was possible that it wouldn't work.” (35 years, no previous children)

#### 3.1.7 Orientation in healthcare system

Women reported a variety of examinations and treatments they had utilised in trying to get pregnant. They expressed the wish to be transferred to a fertility center quickly instead of undergoing lengthy treatment at the obstetrician. Additionally, women wished to get an overview of potential care providers in this setting as well as to receive help in looking for psychological support. Moreover, they mentioned that several treatment options were expensive out-of-pocket services.

“I think it would be good if midwives were already there, when you start to think about having children. Then I would find it helpful just to know who to contact if it doesn't work out.” (24 years, no previous children)

### 3.2 Pregnancy

#### 3.2.1 Care by midwife

During pregnancy, women valued the competencies of midwives, encompassing professional knowledge and a personal relationship. Women wished to be prepared for the upcoming steps and would like the competencies of midwives to be expanded. They asked for continuity of care, meaning that they valued to have the same midwife in different phases (pregnancy, childbirth, postpartum) and asked for regular meetings with their midwife as well as clear communication during substitutions in case another midwife had to step in. They also appreciated having the same midwife throughout different pregnancies. Women wanted to be able to easily reach their midwife. For example, many women valued that they could write text messages to their midwife with her answering as soon as possible. Women appreciated care that was provided at home or in an undisturbed environment (e.g. a midwife practice or a birthing center). Regarding the relationship with their midwife, women asked for empathy, calmness and time, empowerment, trust as well as appreciative and intimate communication. At the same time, some women recognised the necessity to accept when the midwife might not be the right fit. Women would like to have the option to entrust responsibility to the midwife. Some women expressed that they needed midwifery care more during childbirth and puerperium as compared to pregnancy.

“Be it in social skills, be it, as I said, the mental, the physical, there are so many areas that midwives cover in their job, which they ideally pass on to the women.” (31 years, no previous children, 36^th^ pregnancy week)

“And I found it very helpful, for example, that there is always someone, who simply gives you this feeling of security. Who really accompanies you.” (24 years, no previous children, 37^th^ week)

#### 3.2.2 Care by obstetrician

Regarding the care women receive from their obstetrician during pregnancy, they asked for good accessibility of healthcare services and to be able to easily reach their obstetrician. Women expressed the need to be afforded enough time to clarify questions during appointments. They wished for individual treatment and a trustful relationship without being put under pressure. The experiences of women in obstetricians’ treatment differed widely, from feeling well taken care of to experiencing a lack of time and empathy.

“If you have other concerns, I always find that they don't really go into it. But I mean, you know how to help yourself with small things, but if something bad were to happen, of course they would help you, but with questions about pregnancy that you have from time to time, which might not be entirely logical, but which come to mind, you're sometimes a bit dismissed.” (37 years, 1 child, 17^th^ week)

“There are no strangers who palpate you, you know the two doctors and the team, and that's really cool. So I think that's why I feel so well taken care of there, because it's just a really good process and nice people.” (35 years, 1 child, 21^st^ week)

#### 3.2.3 Involvement of family

During pregnancy, women received great support from their partners. At the same time, they wished for their partners to also receive information and be involved in midwifery and obstetric care. Women who already gave birth to a child would like to receive information about how to involve and prepare the firstborn to become a sibling.

“Also that when my husband is there, he can ask questions and is taken seriously.” (31 years, no previous children, 34^th^ week)

“The preparation for the first child also taking on a new role as a sibling, as a big sister, that is definitely something where I am also very happy that my midwife includes this in her birth preparation course.” (30 years, 1 child, 32^nd^ week)

#### 3.2.4 Need for information

Women described a variety of sources for information, namely midwives and obstetricians, but also other experts at counselling centres, hospitals, and health insurance companies. Many women discussed the topic with family, friends, and other mothers or pregnant women, while knowing to be careful with ‘well-intentioned’ advice. Further sources of information were the internet – though seen with scepticism – as well as books and brochures. In some cases, the women's own or their partner's professional background served as a source of information. The topics and questions that arose during pregnancy were manifold, ranging from questions regarding lifestyle and medical issues, such as pregnancy beyond 40 weeks, induction of labour, to organisational questions, preparing oneself for childbirth and the time postpartum as well as preparing the first essential supplies for the child. Regarding the availability of information, women asked for reliable content and reliable sources. They would also like to receive information without having to actively enquire about all topics. They pointed out that a lot of information might be difficult to obtain for underprivileged women, calling for low-threshold information. Women valued a second opinion on certain issues and being well-prepared thanks to interprofessional communication or getting support from multiple sources. However, this could lead to contradictory information, contributing to a feeling of uncertainty. While some women wanted to receive information early on, others preferred to receive information step by step.

“I know that my midwife has a good understanding of scientific research. That she knows which scientific platform I can use, which studies are reputable, which sources are reliable and which are not, that is incredibly important to me.” (30 years, no previous children, 35^th^ week)

#### 3.2.5 Physical aspects

While some women reported having no pregnancy discomforts, others reported a range of different symptoms and complaints. These women wished for improvement of these struggles, to remain mobile and to be prepared for childbirth. They reported a need for advice and would like to have a person to talk to.

“Especially in the first three months or up to the 12th week of pregnancy, I had a few things where I would have liked a bit more support. For one thing, I had extremely intense nausea and didn't really know what to do with it, because I didn't have a midwife until then. So, in general, the way your body changes in the first pregnancy, when you haven't had a child yet, it's strange when your breasts get bigger, when you have constant nausea, I also had a lot of heartburn. And yes, all these things, I just couldn't address them in the complexity in which it occupied my mind all day.” (32 years, no previous children, 14^th^ week)

“Mobility in pregnancy, especially if you like to ride a bike and also ride a bike every day, if you also like to move around a lot, it was really worth its weight in gold to be able to discuss this directly with her, ‘I am in pain, what can I do?’ and ‘yes, come here, lie down on my couch and I will treat you now’.” (30 years, 1 child, 32^nd^ week)

#### 3.2.6 Psychological aspects

Women reported a range of different emotions, that could be directly discussed with a midwife in a timely manner, if necessary. While women looked forward to the arrival of their child, they also expressed worries about the feasibility and what life will be like with (another) child. Women with previous pregnancies described that they could rely on their experiences. Different psychological burdens were mentioned, for example mental exhaustion, crying due to hormonal changes but also concerns regarding the relationship with their partner. Women also expressed concerns for their unborn child. Some women were able to ease their mind by acknowledging their individuality and that every childbirth is different. To take off the pressure, women expressed a sense of *“it'll be alright”,* accepting that they cannot control everything and trying to find a healthy balance.

“I don't really have hormonal fluctuations like that, I've always been joyful and excited, and I am also expecting a planned child. So everything is rather positive for me.” (34 years, no previous children, 16^th^ week)

“And of course fears, so much: ‘God, will I be able to cope as a mother later on?” (31 years, no previous children, 34^th^ week)

#### 3.2.7 Orientation in healthcare system

While some women preferred to receive prenatal care by obstetricians, others made or would have in hindsight wished to make use of the split-care-model in which prenatal care is provided by both midwives and obstetricians. The knowledge about possible care models was very different between participants: while some had received sufficient information about the possibilities and had free choice, others had not received any information about the split-care-model or their obstetricians refused to split prenatal care. Women in alternating care appreciated getting to know the midwife prior to postpartum care, valued that it was a good opportunity to involve siblings, and felt well-informed. The only disadvantage some women mentioned were fewer medical examinations they might receive in the split-care-model. Regarding the utilisation of services and courses, women reported a variety of courses they took part in, ranging from birth preparation courses over gymnastics and acupuncture to courses especially for infant care and multiparae women. However, women criticised the communication in the system with a lack of information transfer between different providers, sometimes resulting in the pregnant woman having to pass on information instead of professionals communicating directly. Finally, women would like to better understand healthcare processes in pregnancy by learning which steps happen when and being informed about/knowing what can be done versus what must be done.

“I also mentioned at the time that I would like to or am thinking about doing the split-care-model, they talked me out of it a bit, let's put it that way.” (33 years, 1 child, 25^th^ week)

“There should be multiple options to tick off, like, this is something I have to do and this is something I can do.” (30 years, 1 child, 32^nd^ week)

#### 3.2.8 Organisation and bureaucracy

Pregnancy comes with a variety of organisational and bureaucratical tasks. This includes the search for a midwife, which was quite difficult for many participants. Women needed to make organisational preparations for childbirth (e.g. acknowledgment of paternity, packing of clinical bag) and the postpartum period (e.g. parental allowance, search for childcare). Considerations about and registration at the birthplace were necessary. Women additionally needed to coordinate pregnancy and other aspects of their lives, especially their jobs.

„I called 36 midwives, sometimes they say, we don't answer the phone, write us an email. That's what I did. And I got 35 rejections and I only found one midwife who was available. And to be honest, I found that extremely unsatisfying.” (32 year, no previous children, 14^th^ week)

“For example, I would also like to go to (specific hospital), but something like that is somehow already booked up, you would have had to take care of it before getting pregnant.” (39 years, no previous children, 19^th^ week)

## 4 Discussion

To our knowledge, this is one of the few qualitative research studies in Germany investigating expectations, preferences and needs of women with the desire to have children and pregnant women in regard to midwifery care. In this study we were able to develop seven main categories in interviews with women in the preconceptional phase and focus groups with pregnant women: (a) care by midwife, (b) care by obstetrician, (c) involvement of family, (d) need for information, (e) physical aspects, (f) psychological aspects and (g) orientation in healthcare system. Through the focus groups with pregnant women, an additional main category evolved: (h) organisation and bureaucracy. Our results are in line with and go beyond the results of the study by Mattern and colleagues [12] who identified three themes regarding knowledge or lack of awareness of midwifery care, availability of and access to midwives, and midwifery care in the healthcare system. These three themes correspond with our categories need for information, care by midwife and orientation in healthcare system, respectively. With our additional main categories, we provided a more extensive perspective including physical and psychological aspects, the involvement of family as an important aspect and the role of the midwife as a key person of trust within the continuity of care. Women's satisfaction regarding comprehensive women-centred care by midwives [17] and a holistic approach are crucial in the improvement of midwifery care [25].

In our study, women were not aware of healthcare services that can be offered by a midwife in the preconceptional phase, due to the fact that such midwifery care and counselling throughout the preconceptional phase is currently seldom provided and the respective competencies of midwives are rarely available or communicated [9]. Thus, women wanting to get pregnant do not know about midwives' role in preconception care. Consequently, they expressed the great need for support in this special phase of life. The category of organisation and bureaucracy seemed to be relevant only for pregnant women and not women with the desire to have children, possibly, due to pregnant women's needs and concerns exceeding the provision of midwifery healthcare services, the changes accompanying pregnancy and preparations for the approaching childbirth. When thinking about antenatal education, pregnant women and/or their partners should complete a prenatal course four weeks before the calculated date of childbirth within the hospital or alternative provider, e.g. the German Red Cross or midwife. However, the access to the antenatal education entails various bureaucratic obstacles.

Dealing with bureaucracy may be suggestive of unequal opportunities for receiving healthcare services. As observed by Bradford and colleagues [3], inequity in the access to midwifery services and healthcare provision is a major concern.

Their research showed that marital status, social status and health literacy of pregnant women and young mothers play a role in the demand for midwifery help and affect the interest in midwifery services and whether they are requested at an early stage [3]. For instance, mothers who are pregnant for the first time and well-educated families take part in birth preparation courses more often than others [5].

To guarantee equal access to healthcare, processes should be standardised. This includes harmonisation of midwifery education in terms of more strongly promoting transfer from practice into the study curriculum and back to practice. Furthermore, midwives would benefit from deriving relevant issues for their curriculum content from the expectations, preferences and needs of study participants. This would enable personalised care and would expand the skill sets of midwives.

During focus groups and interviews with women, we observed how much they benefited from sharing and exchanging their experiences. In the ASSIST project described by Hotton and colleagues, digital instruments and the i-dicide platform (https://europe.idecide.com) have been used to promote women's decision making [26]. By implementing similar exchange platforms, we could support women in Germany in the preconceptional phase and during pregnancy by sharing knowledge and promoting shared decision making as well as learning from international evidence.

Our study has multiple strengths. The substantial advantage and novelty of our study is investigating expectations, preferences and needs of women in regard to midwifery care in the preconceptional phase and thus the conceptual extension of midwifery continuity of care. Conceptually, the usage of mixed methods based on the sequential exploratory design opened up new opportunities for future research in primary healthcare in regard to midwifery care [19]. We therefore consider the qualitative approach to achieve our research aims as another strength of the study, as qualitative methods have been regarded as an opportunity for in-depth insights from study participants and as a basis for quantitative tools. Moreover, according to COSMIN (COnsensus-based Standards for the selection of health Measurement INstruments) [27], a qualitative approach is crucial for PROM (Patient Reported Outcome Measure) development [[17], [28]], and the results of this study will be used as a basis for this. Another strength of our study is the fact that we closed the research gap along the whole continuity of midwifery care, which we observed in midwifery sciences in Germany. Furthermore, our study findings could be implemented in the teaching of midwifery students and used for the development of the curricula nationwide.

We faced a few limitations: Even though we recruited participants nationwide and with the intention of yielding a heterogeneous sample, the greatest share of participants lived in the metropolitan region of Hamburg, in Northern Germany, who were well educated and potentially more interested in the topic than women with a different socioeconomic background. Nonetheless, the sample included perspectives from different subgroups of women. Inherent to the qualitative approach, we cannot quantify the prevalence of different needs. These will be addressed in subsequent research.

Another limitation refers to the coverage of the entire user's perspective, including the preconception phase. As women in our study were not necessarily aware of the role that midwifery care can play in this phase, the wishes and needs they expressed were independent of the scope of midwifery and standard care as currently provided in Germany. When reflecting these individual needs in the care process, it should be considered carefully which tasks could be handled by midwives and which should be handled by other providers such as obstetricians or psychologists.

Some methodological limitations also need consideration. The involvement of multiple researchers (as we enabled for the focus groups) in the data collection and the analysis is essential in qualitative research for data quality and reliability of results. However, for the interviews with women who have the desire to have children, involvement of multiple interviewers was not possible. We considered it essential that a midwife who is experienced and currently active in the counselling interviewed this particularly vulnerable group. Only one researcher in our team met this requirement. Thus, we are aware that the selection of a single researcher in the interviews in our study might influence the results. Another limitation might be the different methods used in the two groups, interviews and focus groups. Despite using two different methods, we aligned categories of both clusters as much as possible in an effort to show similarities and differences within the groups of preconception and pregnancy. From the perspective of the participants, including women who used different care models, e.g. healthcare services provided exclusively by a midwife or solely by an obstetrician, or the combination of both, midwifery care is crucial throughout the entire continuity of care including preconception and pregnancy. Based on our results, midwifery care could be further aligned with the actual needs of women and individualised care implemented for every woman. This would enable the provision of women-centred care [13].

## 5 Conclusions

The present research was designed as a qualitative study using focus groups and interviews, analysing data inductively with the purpose to investigate women's expectations, preferences and needs in midwifery care. In our study, women expressed a variety of different needs throughout the phases of preconception and pregnancy. They especially valued the mixture of professional expertise and personal relationship with midwives, particularly regarding information within the categories need for information, physical and psychological aspects, involvement of family, and orientation in the healthcare system. The variety of needs highlight the interpersonal differences between women and consequently the need for personalised provision of care. Results of the present study will support the development of a tool assessing the quality of healthcare and evaluating women's needs and benefits of midwifery care. With this instrument, we intend to facilitate the adequate depiction of the care situation of women and to personalise care.

## Funding

This research was supported by University Medical Center Hamburg-Eppendorf (UKE) and did not receive any specific grant from funding agencies in the public, commercial, or not-for-profit sectors.

## CRediT authorship contribution statement

**Toni Maria Janke:** Writing – review & editing, Writing – original draft, Validation, Software, Project administration, Methodology, Investigation, Formal analysis, Data curation, Conceptualization. **Nataliya Makarova:** Writing – review & editing, Writing – original draft, Visualization, Validation, Software, Project administration, Methodology, Investigation, Formal analysis, Data curation, Conceptualization. **Janne Schmittinger:** Writing – review & editing, Conceptualization. **Caroline Johanna Agricola:** Writing – review & editing, Conceptualization. **Merle Ebinghaus:** Writing – review & editing, Visualization, Validation, Formal analysis, Conceptualization. **Christine Blome:** Writing – review & editing, Validation, Supervision, Methodology, Investigation, Conceptualization. **Birgit-Christiane Zyriax:** Writing – review & editing, Validation, Supervision, Methodology, Investigation, Conceptualization.

## Declaration of competing interest

The authors Toni Maria Janke, Nataliya Makarova, Janne Schmittinger, Caroline Johanna Agricola, Merle Ebinghaus and Birgit-Christiane Zyriax declare that they have no known competing financial interests or personal relationships that could have appeared to influence the work reported in this paper.

Christine Blome declare the following financial interests/personal relationships which may be considered as potential competing interests: grants or contracts to the institution from Amgen/Celgene, AstraZeneca, Bauerfeind, Pfizer, The EuroQol Group; spreaker honoraria from Amgen/Celgene, AstraZeneca, Hartmann, Helios Klinik Leisnig, medi; support for attending meetings and/or travel from AstraZeneca, Hartmann, Helios Klinik Leisnig.
