# Supplementary material for: Women's needs and expectations in midwifery care – Results from the qualitative MiCa (midwifery care) study. Part 1: Preconception and pregnancy
Source: Heliyon. 2024 Feb 7;10(4):e25862. doi: 10.1016/j.heliyon.2024.e25862 (PMC10867638; doi:10.1016/j.heliyon.2024.e25862)
Supplement: Multimedia component 1 [file mmc1.docx]

**Supplementary Material**

**Women’s needs and expectations in midwifery care – results from the qualitative MiCa study. Part 1: Preconception and pregnancy**

**Box S1** Development of the categories (left, original German wording; right, translation)

| 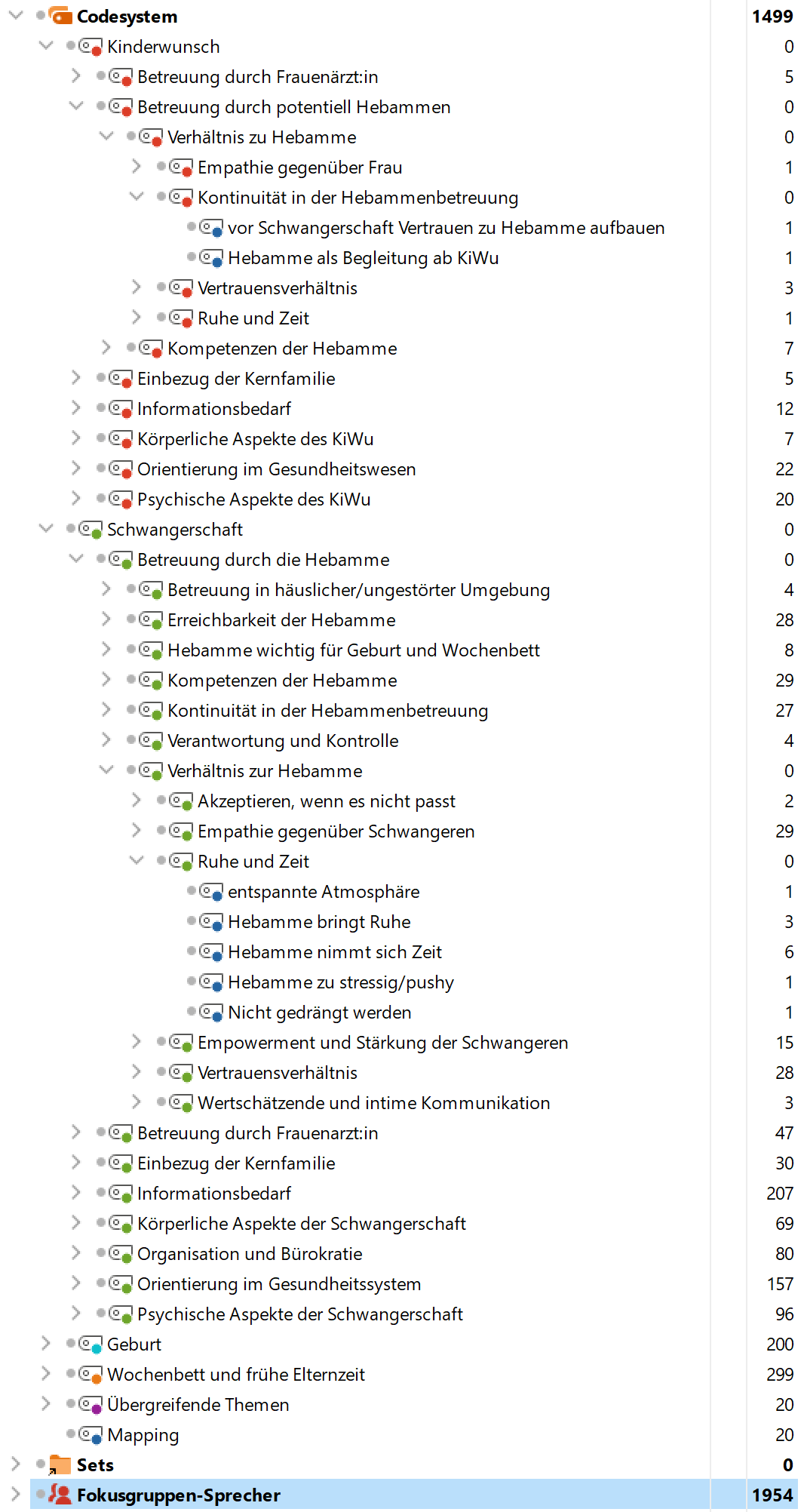 | 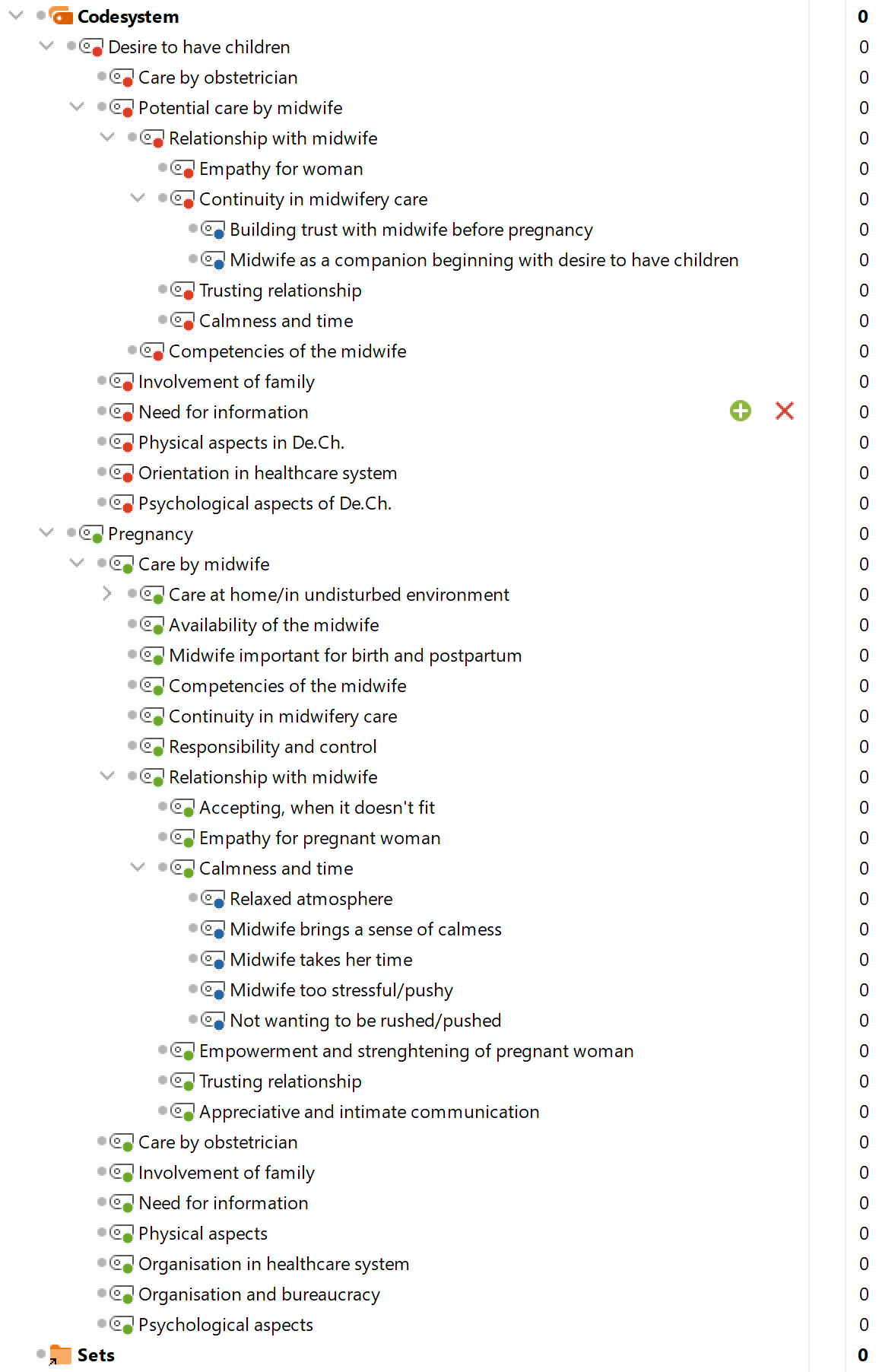 |
| --- | --- |

**Table S1** Consolidated criteria for reporting qualitative studies (COREQ): 32-item checklist

| **No. Item** | **Guide questions/description** | **Reported on Page** |
| --- | --- | --- |
| **Domain 1: Research team and reﬂexivity** | | |
| *Personal Characteristics* | | |
| 1. Interviewer/facilitator | Which authors conducted the interview or focus group? | TMJ, NM, JS, ME |
| 2. Credentials | What were the researcher’s credentials? E.g. PhD, MD | Beginning with Master of Science and ending with Professorship (see title page with affiliations) |
| 3. Occupation | What was their occupation at the time of the study? | All co-authors were occupied as scientific staff at the UKE, p. 7 |
| 4. Gender | Was the researcher male or female? | All authors are female (title page) |
| 5. Experience and training | What experience or training did the researcher have? | TMJ, NM and JS were trained in qualitative research methodology and had experience in conducting qualitative research, p.7-8 |
| *Relationship with participants* |  |  |
| 6. Relationship established | Was a relationship established prior to study commencement? | No relationship was established beforehand. The researchers did not know the study participants before starting the interviews or focus groups, p. 6 |
| 7. Participant knowledge of the interviewer | What did the participants know about the researcher? e.g. personal goals, reasons for doing the research | At the beginning of each interview or focus group, every researcher (TMJ, NM, ME, JS) introduced themselves, their area of research, their role in the research group, and their experience in qualitative research, p. 7.  Participants were briefed on the purpose of the study and the reason for conducting the study. Participants reviewed the participant information documentation prior to giving their written informed consent to be involved, p. 6 |
| 8. Interviewer characteristics | What characteristics were reported about the interviewer/facilitator? e.g. Bias, assumptions, reasons and interests in the research topic | The authors are active in healthcare research and midwifery science, see affiliations |
| **Domain 2: study design** | | |
| *Theoretical framework* | | |
| 9. Methodological orientation and Theory | What methodological orientation was stated to underpin the study? e.g. grounded theory, discourse analysis, ethnography, phenomenology, content analysis | Methodologically, we based our research strategy on the sequential exploratory design by Vedel et. al, p. 5, and used qualitative content analysis according to Mayring (Mayring, 2015), p. 7 |
| *Participant selection* | | |
| 10. Sampling | How were participants selected? e.g. purposive, convenience, consecutive, snowball | Participants were selected purposively, p. 6 |
| 11. Method of approach | How were participants approached? e.g. face-to-face, telephone, mail, email | Through midwives, social media, personal contacts and newsletter at the study center UKE, providing contact information, telephone and email, p. 6 |
| 12. Sample size | How many participants were in the study? | N = 26, Table 1, p. 8 |
| 13. Non-participation | How many people refused to participate or dropped out? Reasons? | The reasons for drop outs were illness or unexpected early delivery, p.6 |
| *Setting* | | |
| 14. Setting of data collection | Where was the data collected? e.g. home, clinic, workplace | Online via Zoom or via telephone, p.7 |
| 15. Presence of non-participants | Was anyone else present besides the participants and researchers? | No |
| 16. Description of sample | What are the important characteristics of the sample? e.g. demographic data, date | Desire to have children, early pregnancy (up to the 24th week of pregnancy) and late pregnancy (beyond the 24th week of pregnancy); age; number of previous children; place of residence; Table 1, p. 8 |
| *Data collection* | | |
| 17. Interview guide | Were questions, prompts, guides provided by the authors? Was it pilot tested? | Researchers experienced in qualitative studies (TMJ and NM) prepared and pilot-tested semi-structured interview and focus group guidelines, p. 7 |
| 18. Repeat interviews | Were repeat interviews carried out? If yes, how many? | No |
| 19. Audio/visual recording | Did the research use audio or visual recording to collect the data? | Interviews and focus groups were audio recorded, p. 7 |
| 20. Field notes | Were ﬁeld notes made during and/or after the interview or focus group? | A second researcher was present in each focus group to document the course of conversation (TMJ, NM or ME), p. 6  JS took notes during the interviews, p. 7 |
| 21. Duration | What was the duration of the interviews or focus group? | Interviews and focus groups took from one hour to 1,5 hours each, p. 7 |
| 22. Data saturation | Was data saturation discussed? | New participants were recruited until the category system reached saturation, meaning that no new themes emerged from additional participants, p. 7 |
| 23. Transcripts returned | Were transcripts returned to participants for comment and/or correction? | No |
| **Domain 3: analysis and ﬁndings** | | |
| *Data analysis* | | |
| 24. Number of data coders | How many data coders coded the data? | Two (NM, TMJ), p. 7 |
| 25. Description of the coding tree | Did authors provide a description of the coding tree? | Box S1 |
| 26. Derivation of themes | Were themes identiﬁed in advance or derived from the data? | Themes were defined in advance by interview and focus group guidelines, p. 7 |
| 27. Software | What software, if applicable, was used to manage the data? | MAXQDA 2022 (VERBI software), p. 7 |
| 28. Participant checking | Did participants provide feedback on the ﬁndings? | No |
| *Reporting* | | |
| 29. Quotations presented | Were participant quotations presented to illustrate the themes/ﬁndings? Was each quotation identiﬁed? e.g. participant number | Results section, pp. 8-15 |
| 30. Data and ﬁndings consistent | Was there consistency between the data presented and the ﬁndings? | Results section, pp. 8-15 |
| 31. Clarity of major themes | Were major themes clearly presented in the ﬁndings? | Table 2, p. 8, figure 1, p. 7 |
| 32. Clarity of minor themes | Is there a description of diverse cases or discussion of minor themes? | Yes, the topic of organisation and bureaucracy during pregnancy in the results section, p. 15 and discussion section, p. 16 |
